# Effects of dietary supplementation of waste date’s vinegar on performance and improvement of digestive tract in broiler chicks 

**Published:** 2017-06-15

**Authors:** Shima Tasharofi, Laleh Yazdanpanah Goharrizi, Farhad Mohammadi

**Affiliations:** 1 *Department of Animal Science Researches, Agriculture and Natural Resources Education and Research Center of Kerman, Kerman, Iran; *; 2 *Graduate Student, Department of Animal Science, Faculty of Agriculture, Shahid Bahonar University of Kerman, Kerman, Iran.*

**Keywords:** Acetic acid, Broiler, Ileum microflora, Jejunum histomorphology

## Abstract

Two hundred 1-day-old commercial broilers (Ross 308) were used to determine the effects of diets supplementation with waste date’s vinegar (WDV) on the growth and performance of digestive tract over a 42-days growing period. Chicks were randomly allocated to one of five experimental diets supplementing as 0 (control), 1, 2, and 3% of WDV and 2% industrial vinegar (IV). Broilers and their feed consumptions were weighed at the trial beginning and days 10, 21, 35 and 42 of experimental period. Moreover, one chick from every replicate was killed at days 21 and 42 to measure development of digestive tissues and morphology and microbiology of small intestine. Although the final body weight was higher following IV and 1% WDV usage, feed conversion ratio was negatively affected by IV usage compared to control (*p *< 0.05). Relative weight of different parts of small intestine was not affected by experimental diets. Villus height and width were reduced linearly in WDV, IV and control groups (*p *< 0.05), but crypt depth was not different among experimental diets. Also, ileum microbiota was not affected by treatments. Results indicated that diet supplementation with WDV has positive effects on growth performance and histomorphology of jejunum in broilers.

## Introduction

Nowadays, efforts have been made to produce high quality animal products without using medicines and reduce environmental contamination by efficient utilization of natural substances. Researchers worldwide are working on organic alternatives due to the ban of a wide range of drugs for animal production. Probiotics consisting of live or dead organisms and spores,^1^ non-traditional chemicals,^2^ bacteriophages,^3^ acidifiers, enzymes and others have emerged in the last decades as some of the tools that could be potentially useful in the near future for pathogen control and poultry performance improvement. Some of these natural substances are not cited in the scientific literature, but are used locally. Such one application in poultry diets, is waste date’s vinegar (WDV) inclusion to diets in south of Iran. Annual production of waste date in Iran, which is not used by human, is 160,000 tones that could be used in poultry diets after removing the kernels. On the other hand, after some processing, WDV could be produced that its main component is acetic acid. Acetic acid is one of the main short chain fatty acids produced by intestinal microbes, which can affect intestinal functions and metabolism.^4^^-^^6^ In addition to these effects of WDV; it includes some beneficial bacteria such as *lactobacillus* spp. that can improve performance, immunity and digestive tract in broiler chicks. A strikingly crucial event in the development of probiotics was the finding that newly hatched chicks could be protected against colonization by *Salmonella enteritidis *through dosing a suspension of gut contents derived from healthy adult chickens which is called competitive exclusion. Also, use of probiotics containing *lactobacilli* offers *S. enteritidis *and *E. coli *growth inhibition in broilers.^7^ Waste date’s vinegar’s *lactobacillus *as a probiotic could be settled in small intestine and as a result, causes infectious bacteria such as *Salmonella* and *E. coli *reduction*. *The intestine seems to be the most fundamental organ for improving animal products. Activation of intestinal function of broilers might increase the meat products in response to an increasing demand for animal protein.^8^ Therefore, it was interesting to investigate how intestinal histology would be affected after WDV feeding. In this study, effects of dietary WDV on body weight gain (BWG) and feed intake (FI) and efficiency were examined in broiler chicks. Then, weight and length of different tissues of digestive tract were measured. Also, jejunum villus height and crypt depth were measured and amounts of ileum *lactobacillus* and *E. coli *were counted.

## Materials and Methods

The experiment was conducted at a commercial broiler farm in Kerman, Iran. Kerman has located at dry and arid area with average annual rainfall of 200 mm and maximum annual temperature of 40 ˚C with height of 1500 to 2000 m. The experimental protocols were reviewed and approved by the Animal Care Committee of Research Institute of Animal Science, Karaj, Iran.

**Preparation of WDV. **Almost one ton of fresh waste date in a commercial vinegar-making workshop in Kerman province was soaked in water, reduced to pulp; the kernels were removed and then, combined with water in ratio of approximately 1:3 waste date and water respectively to produce WVD before being used in the present study.

**Chemical and microbial analyses. **Representative samples of WDV and industrial vinegar (IV) were analyzed for percentages of acetic acid (titration method with a colored pH indicator) which were 2.60 and 10.40, respectively. Also, samples of WDV were tested for microorganism existence (microbial culture method) which showed lactobacillus, bacillus and mold existence with total account of 4.50 × 10^5^ cfu. Table 1 shows the chemical and microbial composition of IV and WDV. Also, it is noticeable that to ignore the effects of different percentages of acetic acid in two vinegars,^9^ during all experiment, IV was four times diluted with distilled water before use.

**Animals, diets and experimental design.** Two hundred one-day-old mixed sex broilers (Ross 308) weighing 40.00 ± 1.50 g were allocated to five experimental diets in a balanced completely randomized design (n = 4) with 20 pens (1.50 × 0.70 m^2^ each) and 10 chicks in each pen. Experimental diets were supplemented using incremental levels of WDV including 0 (control), 1, 2 and 3% of diet and 2% of IV. Table 1 presents the chemical composition of diet^10^ and all chicks had free access to feed and water *ad-libitum*. Chicks were raised under similar environmental conditions based on Ross 308 management recommendations for 42 days.^11^

Experimental period lasted 42 days. The feed amounts offered and refused were measured periodic at days 10, 21, 35 and 42 for each pens to calculate FI. Moreover, the chicks were weighed at these days after 2 hr of fasting to reduce the disputes arising from feed consumption and these weights were used to calculate the body weight (BW) changes and average periodic weight gain of chicks over the experimental time. By having these two measure-ments, feed conversion ratio (FCR) was calculated as feed consumed per unit of gain. Before the beginning of experiments, all animals were vaccinated for bronchitis and routine vaccinations including Newcastle (at days 8, 17 and 28) and Gambro (at days 13 and 24) were done during the growing period. Chicks were visited daily in a regular program for general health and some individual behaviors including illness, breath and anorexia.

**Digestive tract sampling and analysis. **At days 21 and 42 of experimental period, four chicks from every treatment (from two pens: male, and from two pens: female, with average weight about 20 g) were sacrifices by cervical dislocation to measure relative weight of different parts of small intestine including duodenum, jejunum and ileum as organ weight (g) / live body weight (g) as well as morphology and microbiology of jejunum and ileum. 

**Jejunum morphology and analysis. **For histopathological and morphometric analysis, 0.50 cm tissue samples from the jejunum of above-mentioned chicks were obtained and fixed in 10% buffered formalin (100 mL of 40% formaldehyde, 4 g phosphate, 6.50 g dibasic sodium phosphate and 900 mL of distilled water) for 24 hr and then, 10% buffered formalin was renewed. Tissues were dehydrated by transferring through series of alcohols with increasing concentrations, placed into xylene and embedded in paraffin. A microtome was used to make five cuts that were 5 μm. The paraffin sections were stained with hematoxylin and eosin.^12^ The values were measured with a light microscope (model DM 1000 LED; Leica Microsystems GmbH, Wetzlar, Germany) using a software (LEICA Queen 550; Leica Microsystems GmbH). Measurements of villus height and width and crypt depth were determined at a magnification of 10×. A minimum of five measurements per slide were made for each parameter and averaged into one value.

**Analysis of ileum microflora. **Digesta were obtained from ileum of above-mentioned chicks and collected in sterile bags to count *lactobacillus* and *E. coli*. Digesta samples were homogenized with 1 mL normal saline. Aliquots (5 μL) were mixed with blood agar and eosin methylene blue and incubated at 37 ˚C for 24 hr. Then, bacteria colonies were counted in selective agar media for enumeration of target bacterial groups. The microbial counts were determined as cfu per gram of wet samples.^13^

**Table 1.** Ingredients and chemical composition of diets and chemical and microbial composition of vinegars

**Ingredients of diet (%)**	**Diets per days**			
	**1-10**	**11-21**	**22-35**	**36-42**
**Corn meal**	58.40	60.40	64.70	66.20
**Soybean**	35.50	33.50	30.20	28.70
**Commercial concentrate** 1	6	6	5	5
**Salt**	1	1	1	1
***Chemical composition of diet (%)***				
**ME (Kcal kg** ^-1^ **)**	2875	2900	2950	2963
**Crude protein**	21.80	21.10	19.80	18.70
**Calcium (%)**	1.25	1.25	1.05	1.05
**Absorbable phosphorous**	0.57	0.56	0.49	0.49
**Sodium**	0.18	0.18	0.16	0.14
**Lysine**	1.30	1.25	1.14	1.10
**Methionine**	0.49	0.48	0.43	0.43
**Methionine-Cysteine**	0.85	0.80	0.75	0.72
**Tryptophan**	0.26	0.26	0.25	0.25
***Chemical and microbial composition of vinegars***		***Kind of vinegars***		
**Acid acetic (g 100 mL** ^-1^ **)**		IV	10.40	
**Acid acetic (g 100 mL** ^-1^ **)**		WDV	2.60	
**Total Count (cfu g** ^-1^ **)**		WDV	4.50 × 10^5^	

1 Made by Arshia Sepehr Co. in Tehran, Iran; IV: Industrial vinegar; WDV: Waste date’s vinegar.

**Statistical analysis. **Data obtained from performance (BWG per period, FI per period and FCR) were analyzed using repeated measurements model in which the time series (1-10, 11-21, 22-35 and 36-42) covariance structure was modeled by using four different covariance structures for each variable tested and the means were compared using Tukey’s multiple comparisons procedure. Other variables based on a completely randomized design were statistically analyzed using GLM procedure in SAS (version 9.1; SAS Institute, Cary, USA) and the means were compared using Duncan’s multiple comparisons procedure. Statistical models were as follows:

*Dependent variable (Y*_ijk_ *) = μ+T*_i_ *+P*_j_ *+T*_i_ *×P*_j_ *+e*_ijk_

and Completely randomized design: 

*Y*_ij_ *= μ+T*_i_ *+e*_ij_

where, *μ* is overall mean, *T*_i_ is treatment, *P*_j_ is period effect and *e*_ij_ is random error.

## Results

**Animal performance. **Data of performance variables are presented in Table 2. The final BW was increased by usage of IV and 1% WDV in comparison to control (*p *< 0.05) but average periodic weight gain was not different between treatments. Feed intake was reduced in control group as compared with 1% WDV treatments (*p *< 0.05) but FCR was negatively affected by use of IV in the diets as compared with control (*p *< 0.05).

**Small intestinal growth.** Table 3 shows growth (relative weight) of different parts of small intestine. All data (relative weights of duodenum, jejunum and ileum) showed no significant difference between treatments. 

**Table 2:** Effect of industrial vinegar (IV) and different levels of waste date’s vinegar (WDV) on performance of broilers

**Parameters**	**Groups**					***p*** **-value**				**SEM**
	**Control**	**2%IV**	**1%WDV**	**2%WDV**	**3%WDV**	**Treat**		**Period**	**Treat × Period**	
**Final BW**	2128.40^b^	2226.00^a^	2250.20^a^	2201.40^ab^	2161.40^ab^	0.050				28.546
**BWG**	521.53	545.96	552.13	539.92	529.66	0.053	< 0.0001		0.006	10.079
**FI**	945.40^b^	1023.81^ab^	1037.58^a^	991.95^ab^	997.19^ab^	0.055	< 0.0001		0.012	29.194
**FCR**	1.84^b^	1.97^a^	1.91^ab^	1.87^ab^	1.92^ab^	0.040	< 0.0001		0.018	0.039

BW: body weight; BWG: body weight gain per period; FI: feed intake per period; FCR: feed conversion ratio.

ab Means within a row with different subscripts differ significantly (*p *< 0.05).

**Table 3:** Effect of industrial vinegar (IV) and different levels of waste date’s vinegar (WDV) on relative weight (g/g) × 100 of different parts of small intestine of broilers

**Parameters**	**Treatments**					***p*** **-Value**	**SEM**
	**Control**	**2%IV**	**1%WDV**	**2%WDV**	**3%WDV**		
***21*** ^st^ *** day***							
**Relative weight of duodenum **	1.38	1.59	1.41	1.33	1.31	0.38	0.0010
**Relative weight of jejunum **	2.61	2.69	2.53	2.48	2.23	0.60	0.0020
**Relative weight of ileum **	2.08	2.27	1.96	2.11	1.82	0.23	0.0013
***42*** ^nd^ *** day***							
**Relative weight of duodenum **	0.82	0.89	0.72	0.72	0.70	0.16	0.0006
**Relative weight of jejunum **	1.75	1.94	1.66	1.87	1.60	0.25	0.0011
**Relative weight of ileum **	1.44	1.46	1.28	1.46	1.26	0.48	0.0010

ab Means within a row with different subscripts differ significantly (*p *< 0.05).

**Table 4:** Effect of industrial vinegar (IV) and different levels of waste date’s vinegar (WDV) on jejunum morphology and ileum microflora of broilers

**Parameters**	**Treatments**					***p*** **-Value**	**SEM**
	**Control**	**2%IV**	**1%WDV**	**2%WDV**	**3%WDV**		
***21*** ^st^ *** day***							
**Villus height (µm)**	989.53^b^	1070.11^b^	1199.80^a^	1268.00^a^	1177.96^a^	0.0002	32.873
**Villus width (µm)**	125.96^c^	176.53^b^	199.42^a^	200.32^a^	195.52^ab^	0.0001	6.937
**Crypt depth (µm)**	193.80	197.63	212.23	197.95	219.96	0.87	20.462
**Villus height : Crypt depth**	5.15	5.53	5.80	6.62	5.72	0.60	0.646
***42nd day***							
**Villus height (µm)**	1188.16^b^	1248.26^ab^	1293.70^a^	1331.69^a^	1258.91^ab^	0.049	30.587
**Villus width (µm)**	175.25^b^	207.65^a^	213.09^a^	225.62^a^	200.87^ab^	0.018	9.381
**Crypt depth (µm)**	246.31	228.55	233.97	239.06	273.77	0.47	18.438
**Villus height : Crypt depth**	4.96	5.63	5.48	5.69	4.72	0.53	0.477
***21st day***							
***Lactobacillus*** ** (Log cfu g** ^-1^ **)**	4.61	4.69	4.81	4.88	4.53	0.42	0.139
***E. coli*** ** (Log cfu g** ^-1^ **)**	4.69	4.17	3.97	3.84	4.42	0.30	0.295
***42*** ^nd^ *** day***							
***Lactobacillus*** ** (Log cfu g** ^-1^ **)**	4.46	4.56	4.70	4.84	4.34	0.42	0.193
***E. coli*** ** (Log cfu g** ^-1^ **)**	4.73	4.22	4.05	3.97	4.47	0.39	0.299

abc Means within a row with different subscripts differ significantly (*p *< 0.05).

**Jejunum histomorphology.** Table 4 shows the intestinal morphology characteristics of broilers including villus height and width, crypt depth and ratio of villus height to crypt depth. Villus height and width increased linearly in control, IV and WDV treatments at 21^st^ and 42^nd^ day of growing period (*p *< 0.05) which showed the beneficial impression of WDV use on villus height and width of broilers. Crypt depth and ratio of villus height to crypt depth were not affected by feeding of experimental diets.

**Ileum microbiota. **According to Table 4, none of ileum microflora variables (including *lactobacillus* and *E. coli* spp. count) were affected by feeding of experimental diets, but WDV and IV supplementation maintained populations of unprofitable bacteria or potential pathogens (*E. coli*) at relatively low levels (numerically) in the ileum’s digesta. Furthermore, *Salmonella* spp. were not detected in content of ileum of broilers while sampling days over the entire of the experimental period.

## Discussion

The performance data are in line with results which reported significantly increase in final BW and improve-ment of FCR by feeding vinegar (including 5% acetic acid) and probiotics in broilers^14^ and better BW and FCR in broilers fed organic acids,^15^ but some have reported that supplementing diet by bamboo vinegar solution does not affect the final BW, FI and FCR of ducks.^8^ Feed intake in chicken is a function of nutritive requirements but on the other hand, supplementing diet by both industrial and waste date vinegar causes the incensement of palatability of feed which may lead to increment of FI.^16^^,^^17^ Based on this theory, in this experiment, all groups containing vinegar have increased FI and positively affected final BW.

Acetic acid in vinegar reportedly increases gastric proteolysis and improves digestibility of proteins and amino acids.^18^ In addition, this acid inhibits growth of harmful intestinal bacteria which compete with the host animal for available nutrients.^19^ The combination of acetic acid and probiotic in WDV is the main reason which controls the balance of intestinal microflora and positively affects intestinal functions and metabolism compared with IV.

The results of small intestine growth are in line with the findings observed no changes in relative weight of different parts of small intestine of broilers derived from probiotic supplementation in diet.^20^ On the other hand, use of butyric acid^21^ and acetic acid^22^ in broiler diet caused an increase of relative weights of jejunum and ileum of broilers. 

Jejunum histomorphology analysis results are in agreement with an increase of jejunum villus height and area on male broilers fed bamboo vinegar liquid.^8^

It is well demonstrated that organic acid in vinegar increases the solubility of nutrients and improves the gastric proteolysis which develops digestibility of proteins and amino acids^18^ and probiotic produces antimicrobial substances and protects the villi and absorption surface against toxins^23^ and mainly promotes secretion of digestive enzymes^24^ that all of these reasons lead to increase in the absorption of available nutrients, a mechanism that directly affects the recovery of the intestinal mucosa, increasing villus height and better intestinal function.^23^

Our findings about the ileum microbiota were not confirmed with the results reported decrease of adverse bacteria in gut microflora of broilers by use of probiotics^25^^,^^26^ and decrease of gram negative bacteria following probiotic and organic acid usage in broiler chicks.^27^ It is generally documented that there are two basic mechanisms by which probiotics act to maintain a beneficial microbial population including competitive exclusion and immune modulation. Competitive exclusion involves competition for substrates, production of antimicrobial metabolites that inhibit the pathogens and competition for attachment sites.^28^ Also, through direct interaction with gut mucosal immune system, probiotics can modulate either innate or acquired immunity, or both to protect the increase in amount of pathogens in gut.^29^

In conclusion, results from current experiment indicate that usage of 1%WDV and IV increases final BW of broiler chicks but IV has adverse effect on FCR. In addition, intestinal morphology is improved by adding WDV to diet of broilers. In conclusion, WDV can be supplemented to diets of broilers to improve growth performance.
